# Ruminococcus gnavus Bacteremia in a SARS-CoV-2 Patient With Diverticulosis

**DOI:** 10.7759/cureus.78784

**Published:** 2025-02-09

**Authors:** Raquel Flores, Paula A Calvo, Constanca Antunes, Rodrigo Duarte, Graça Lérias

**Affiliations:** 1 Internal Medicine, Hospital de Egas Moniz, Lisbon, PRT; 2 Endocrinology, Diabetes and Metabolism, Hospital de Egas Moniz, Lisbon, PRT; 3 Infectious Diseases, Unidade Local de Saúde de Lisboa Ocidental (ULSLO), Lisbon, PRT

**Keywords:** diverticulosis complications, geriatric medicine, infectious disease pathology, ruminococcus gnavus, sars-cov-2

## Abstract

*Ruminococcus gnavus* is a constituent of the human intestinal microbiota, found in the commensal flora of healthy individuals. Changes in the intestinal microflora associated with chronic conditions and immunosuppression promote the bacterial translocation of *R. gnavus*.

We present a case of bacteremia due to *R. gnavus* in an elderly man with multiple comorbidities, including diverticular disease and moderate SARS-CoV-2 infection requiring corticosteroid therapy. He had a prolonged hospital stay and multiple infectious complications.

According to the literature review in databases such as PubMed (the last search was conducted in August 2023), a total of 17 cases were described. The reported cases had in common gastrointestinal symptoms such as gastrointestinal bleeding, diverticular disease, ulcerative colitis, cholecystitis, gastrointestinal fistula, or infection due to an orthopedic prosthesis. There was also a case in a patient with hemato-oncological disease, previously treated with a cycle of corticosteroids.

It was considered that, alongside the history of diverticular disease, the immunosuppression secondary to the corticosteroid therapy for the SARS-CoV-2 infection may have contributed to the imbalance of the intestinal microbiome, leading to the occurrence of bacteremia due to this commensal agent.

## Introduction

*Ruminococcus gnavus* was first identified in 1974 as a strict anaerobe of the human GI tract. It is a Gram-positive anaerobic bacterium, belonging to the phylum Firmicutes and the class Clostridia, a constituent of the intestinal microbiota [1].

It is often found in the commensal flora of the intestine and is present in the gut of 90% of people. Nonetheless, it represents less than 0.1% of the intestinal microbiota in healthy individuals.

*R. gnavus *has mucolytic activity due to the action of glycosidase, facilitating its contact with the intestinal epithelium. Its beta-glucuronidase activity allows it to generate toxic metabolites and potential carcinogens, thus causing inflammation [2].

Changes in the intestinal microflora are associated with several pathologies, namely inflammatory bowel disease (IBD), *Clostridium difficile* infection, spondyloarthritis, and atopic eczema [3,4].

The authors report the first case of *R. gnavus* bacteremia associated with both COVID-19 disease and the immunosuppressive effects of its treatment.

## Case presentation

We present a case of an 89-year-old male with a medical history of diverticular disease, chronic obstructive pulmonary disease, prostatic cancer medicated with leuprorelin, atrial fibrillation medicated with a direct oral anticoagulant, gout, dyslipidemia, hypertension, nephrolithiasis, and a history of myoepithelial parotid neoplasm and thyroid neoplasm submitted to surgical excision of these glands. The patient presented to the ED due to confusion and temporo-spatial disorientation of gradual onset, with four months of evolution and aggravated in the four days prior to hospital admission, with no known cause, loss of consciousness, or other symptoms.

The initial observation was relevant for the patient’s disorientation in time, space, and self, with normal vital signs, bilateral crackles at pulmonary auscultation, and bilateral edema of the lower limbs.

The blood analysis showed normochromic normocytic anemia (hemoglobin (Hb) 10.7 g/dL, mean corpuscular volume 92.2 fL, mean corpuscular hemoglobin concentration (MCHC) 330 g/L), leukocytosis of 10,400 cells/uL with neutrophilia of 88%, C-reactive protein 23.1 mg/dL, with dicumarinic intoxication with an international normalized ratio (INR) of 7.9 and an activated partial thromboplastin time of 49.6 s. The arterial blood gas sample evidenced hypoxemic respiratory failure, partial oxygen pressure of 56.2 mmol/L, and a peripheral saturation of 89% at room air (Table 1).

**Table 1:** Laboratory results. aPTT: Activated Partial Thromboplastin Time; Cr: Creatinine; fl: Femtoliters; g/dL: Grams per Deciliter; HCO3: Bicarbonate; INR: International Normalized Ratio; MCV: Mean Corpuscular Volume; mg/dL: Milligrams per Deciliter; mmol/L: Millimoles per Liter; pCO2: Partial Pressure of Carbon Dioxide; pO2: Partial Pressure of Oxygen; PCT: Procalcitonin; RCP: Reactive Chain Protein; s: Seconds; μl/L: Microliter per Liter.

Laboratory studies	Results	Reference values
pH	7.44	7.35-7.45
pCO2 (mmHg)	40.8	35.0-45.0
pO2 (mmHg)	80.1	75.0-100.0
HCO3 (mmol/L)	27.0	21-28
Lactate (mmol/L)	1.40	0.50-2.00
Hemoglobin (g/dL)	10.7	13.0-17.0
Hematocrit (%)	32.5	40.6-50.4
MCV (fL)	92.2	80.0-96.1
WBC (cells/L)	10.4 x 10^9^	4.0-10.0 x 10^9^
Neutrophils (%)	88.9	40.0-80.0
Platelets (cells/L)	189 x 10^9^	150-400 x 10^9^
INR	7.9	(according to the pathology)
aPTT (s)	49.6	23-38
Cr (mg/dL)	1.04	0.70-1.20
Urea (mg/dL)	69	17-49
Sodium (mmol/L)	146	136-145
Potassium (mmol/L)	4.39	3.50-5.10
RCP (mg/dL)	23.1	<0.5
PCT (ng/mL)	0.14	<0.1

Due to the epidemiologic context, a SARS-CoV-2 swab was obtained, which was positive. The chest X-ray showed bilateral interstitial infiltrate and the cranial CT scan was relevant for chronic microangiopathic ischemic leukoencephalopathy.

It was assumed a moderate COVID-19 pneumonia and dicumarinic intoxication. The patient was hospitalized in an isolation room, undergoing a cycle of five days of dexamethasone 6mg/day, with a good initial clinical evolution. During his quarantine, he presented several complications. Firstly, a nosocomial urinary tract infection due to Proteus mirabilis, medicated according to its antibiotic susceptibility testing, and concomitantly, aggravated anemia (Hb 7.5 g/dL) with no known blood losses, with an INR of 5.84 on warfarin. Endoscopic exams were requested, showing non-erosive gastropathy and a colonoscopy not conclusive due to poor intestinal preparation.

Despite an eight-day antibiotic cycle with piperacillin/tazobactam, due to persistent fever and elevated inflammatory markers, blood and urine cultures were requested. The blood cultures were positive for pan-susceptible *R. gnavus* and methicillin-resistant *Staphylococcus aureus*, and the urine culture was positive for *Enterococcus faecium*. The patient was then initiated on vancomycin. A decline in the patient’s respiratory function was observed, necessitating non-invasive ventilation. A thoracic angiographic CT scan was requested, showing ground-glass consolidation on the left lung's superior lobe.

He was given two days of 40 mg of methylprednisolone due to suspected organizing pneumonia. The treatment was suspended after multidisciplinary discussion because of a lack of clinical and radiological criteria for its treatment. A respiratory sputum culture was obtained, showing MRSA, motivating a total of 14 days of vancomycin. After the antibiotic treatment, new cultures were obtained, which came back negative. A clinical recovery was observed and the patient was discharged clinically better.

## Discussion

We report the seventeenth clinical case associated with bacteremia due to *R. gnavus*, the first in a patient with diverticular disease and moderate COVID-19 pneumonia, with a need for corticotherapy (dexamethasone).

Table 2 summarizes the clinical features of all 17 cases, revealing a heterogeneous population (8 male, 9 female). Gastrointestinal pathology was the most prevalent characteristic (13/17, 76.5%), suggesting bacterial translocation from the gut as a primary infection pathway. Fever was also common at presentation.

**Table 2:** List of Ruminococcus gnavus clinical cases and their main features.

Gender	Age	Oncologic records	Corticoid therapy	Surgical records	GI comorbidity	Isolation	Symptoms	Reference
F	66	None	No	Hernioplasty, cholecystectomy, hysterectomy	Intestinal perforation	Blood culture	Hypotension, tachycardia, abdominal pain, anorexia, vomiting	Lefever S et al. [5]
M	67	Lung small cell carcinoma	No	No	Perforated diverticulitis	Blood culture	Abdominal pain, fever.	Hanse Hl et al. [6]
M	90	None	No	No	Diverticulitis	Blood culture	Abdominal pain, vomiting, fever.	Hansel H et al. [6]
M	47	Unidentified primary squamous carcinoma	No	No	Possible gastrointestinal fistula	Joint fluid culture	Right hip pain, fever.	Titécat M et al. [7]
M	62	None	No	Arthroplasty	Active ulcerous colitis	Bone biopsy	Right hip pain, fever	Roux AL et al. [8]
F	93	None	No	Arthroplasty	No	líquido post sonicação prótese retirada	Inguinal pain.	Arnáez Solís R et al. [9]
F	72	None	No	Arthroplasty	No	Cultura tecido periarticular	Inguinal pain, fever, intermittent claudication	Fernández-Caso et al. [10]
Not clarified	Not clarified	None	No	No	Strangulated hernia	Blood culture	Not revealed.	Struyve M et al. [11]
F	27	None	No	Laparoscopy for bilateral tubo-ovarian abscesses	No	Cultura material purulento de abcesso tubo-ovárico recidivante.	Abdominal pain, fever, anorexia	Veale R et al. [12]
F	82	None	No	Endoscopic retrograde cholangiopancreatography (ERCP) and percutaneous trans-hepatic gallbladder drainage	Perforated cholecystitis and choledocholithiasis	Blood culture	Fever, abdominal pain, cough with sputum	Kim Y et al. [13]
F	76	Multiple myeloma and myelodysplastic syndrome	Yes	Rotator cuff surgery	Lower digestive hemorrhage, C. Difficile infection 2 month prior	Blood culture	Asymptomatic	Gren et al. [1]
M	85	None	No	No	Cholelithiasis	Blood culture	Fever, mouth ulcers	Xin Fan et al. [14]
F	74	Cervical cancer	No	Percutaneous pelvic abscess drainage due to bladder perforation	No	Blood culture	Fever	Furutani T et al. [15]
F	67	None	Yes	Liver transplantation	Ulcerous colitis	Blood culture	Fever	Martínez de Victoria Carazo J et al. [16]
M	73	None	Yes	No	No	Blood culture	Abdominal pain	Hioki T et al. [17]
M	77	Multiple myeloma, colorectal cancer	No	No	Colorectal cancer	Blood culture	Septic shock, multiple organ failure	Fontanals D et al. [18]
M	89	Prostatic adenocarcinoma, thyroid neoplasm	Yes	No	Diverticulitis	Blood culture	Fever, respiratory distress	Present case

In the presented case, a hemoglobin drop of unclear etiology occurred after a short course of corticosteroids. *R. gnavus* bacteremia was identified on hospital day 34, after the colonoscopy. This, coupled with the patient’s history of diverticulitis and lower gastrointestinal bleeding, supports a diagnosis of gut bacterial translocation secondary to compromised intestinal wall integrity, exacerbated by corticosteroid use.

As such, the authors believe that the conjunction of altered intestinal microflora (dysbiosis) associated with diverticular disease and the immunosuppression due to the SARS-CoV-2 infection and its treatment, allowed for the isolation of *R. gnavus* in blood cultures.

## Conclusions

The presented case attributes the bacteremia caused by *Ruminococcus gnavus *to the association of intestinal microbiome dysbiosis with oxidative stress. The conjunction of altered intestinal microflora, associated with diverticular disease, and the potentiation of immunosuppression due to the SARS-CoV-2 infection and its treatment, allowed for its isolation in blood cultures. Despite being a commensal agent, it has pathogenic potential in states of immunosuppression. Increased sensitivity and concern are essential when isolating less common agents such as *R. gnavus*. Additional investigations are needed to better understand the role of this agent in immunosuppressive states.
